# Efficacy of Immune Checkpoint Inhibitors in Patients With EGFR Mutated NSCLC and Potential Risk Factors Associated With Prognosis: A Single Institution Experience

**DOI:** 10.3389/fimmu.2022.832419

**Published:** 2022-02-28

**Authors:** Menglin Bai, Weiqing Wang, Xuetian Gao, Leilei Wu, Peng Jin, Hui Wu, Jinming Yu, Xue Meng

**Affiliations:** ^1^Department of Radiation Oncology, Shandong Cancer Hospital and Institute, Cheeloo College of Medicine, Shandong University, Jinan, China; ^2^Department of Radiation Oncology, Shandong Cancer Hospital and Institute, Shandong First Medical University and Shandong Academy of Medical Sciences, Jinan, China; ^3^Department of Radiation Oncology, Shanghai Pulmonary Hospital, School of Medicine, Tongji University, Shanghai, China; ^4^Department of Oncology, Affiliated Cancer Hospital of Zhengzhou University, Zhengzhou, China

**Keywords:** epidermal growth factor receptor (EGFR), immune checkpoint inhibitor (ICI), non-small cell lung cancer (NSCLC), efficacy, prognosis

## Abstract

**Background:**

The role of immune checkpoint inhibitors (ICIs) in NSCLC patients with EGFR mutations are controversial. In this study, we aim to investigate the therapeutic efficacy of ICIs alone or in combination in patients with EGFR mutated NSCLC in late-line settings, and explore the factors that may predict the efficacy of ICIs.

**Patients and Methods:**

We retrospectively collected the clinical and pathological information of 75 patients with confirmed EGFR mutations. All patients have developed acquired resistance to EGFR-TKIs, and were treated with ICIs in late line settings from January 2019 to January 2021, at Shandong Caner Hospital and Institute. Therapeutic efficacy was evaluated by tumor response and survival.

**Results:**

The median follow-up period was 7.3months (range 1.8-31.8 months). The overall response rate (ORR) was 8.0%, and the disease control rate (DCR) was 78.7%. The median PFS for all patients was 3.9 months (95% CI, 2.7-5.0), while the median OS was 9.9 months (95% CI, 5.3-14.6). We found that patients with longer response duration to EGFR-TKIs (≥10 months) showed a longer PFS when treated with immunotherapy compared with patients with shorter PFS-TKI (<10 months), the median PFS in two groups were 5.2 months [95%CI 4.2-6.2] and 2.8 months [2.0-3.6]) respectively (HR, 0.53, 95%CI, 0.31-0.91, *P*=0.005). In exploratory analysis, we found that concurrent extracranial radiotherapy and higher body mass index (BMI) are associated with longer PFS (*P* values are 0.006 and 0.021 respectively).

**Conclusions:**

We found that combination regimen of immunotherapy plus chemotherapy plus antiangiogenetic agents may yield longer survival in patients with EGFR mutated NSCLC. We also found that patients with longer PFS-TKI, concurrent extracranial radiotherapy and higher BMI may benefit more from immunotherapy.

## Introduction

Epidermal growth factor receptor (EGFR) mutations remains the most common driver mutations in patients with lung adenocarcinomas (LUAD), with an incidence of 50% in Asians and 9.8% in Caucasian Europeans (1, 2). The development of EGFR tyrosine kinase inhibitors (EGFR-TKIs) have significantly prolonged the survival of patients harboring EGFR mutations. The progression-free survival (PFS) of first- and second-generation EGFR-TKIs, including Gefitinib, Erlotinib, Icotinib, and Afatinib, is usually around 9-13 months, while the third generation EGFR-TKI, Osimertinib, yielded a PFS of 18.9 months. Despite these progress, drug resistance and disease progression are inevitable. Hence, disease management after TKI-resistance has become a critical issue, and require further studies.

Immune checkpoint inhibitors (ICIs) are group of monoclonal antibodies targeting immune checkpoints including PD-1/PD-L1 and CTLA-4 etc. By blocking the interaction between immune checkpoints and their partners, ICIs mediate tumor killing effects through unleashing the “breaks” of immune system (3). Although ICIs, especially those targeting PD-1 and PD-L1 has been proven to be effective in patients with advanced NSCLC, their roles in patients harboring EGFR mutations are still in debate. Pre-clinical evidences suggested that activation of EGFR would up-regulate the expression of PD-L1 through numerous signaling pathways, including p-ERK1/2/p-c-Jun and JAK/STAT3, and blockade of PD-1 would improve the survival of murine models with EGFR mutated lung adenocarcinomas by promoting T cell infiltration and down-regulating pro-tumorigenic cytokines (4–6). However, it is well-demonstrated that ICIs alone or in combination with TKIs could not yield improvements in survival in patients with EGFR mutations compared with general unselected NSCLC patients (7–10).

Luckily, recent studies have shed some light into this area. In PROLUNG trial, which compared the therapeutic efficacy of pembrolizumab plus docetaxel versus docetaxel alone in pretreated NSCLC patients, 25 patients with EGFR mutations were enrolled. The PFS in patients received pembrolizumab plus docetaxel was significantly prolonged compared with patients received docetaxel alone. The more recent IMpower150 study further uncover the value of anti-angiogenic therapy in EGFR mutated NSCLC. Patients received atezolizumab and bevacizumab plus chemotherapy had better clinical outcome with an ORR of 73.5% (versus 40.9% in bevacizumab plus chemotherapy group) and median PFS of 10.2 months (versus 7.1 months in bevacizumab plus chemotherapy group) (11, 12). However, real-world efficacy of ICIs in pretreated EGFR mutated NSCLC patients remains limited (13, 14).

In this retrospective study, we aim to examine the therapeutic efficacy of ICIs alone or in combination in patients with EGFR mutated NSCLC in real-world setting. We also explore the potential clinical and pathological characteristics that may predict the efficacy of ICIs.

## Patients and Methods

### Study Design and Patients

In this retrospective study, we aim to study NSCLC patients with EGFR mutations treated with immune checkpoint inhibitors after receiving EGFR-TKIs. All patients are enrolled at Shandong Cancer Hospital and Institute, form January 2019 to January 2021. The inclusion criteria were as follows: 1) stage IIIB-IVB NSCLC with confirmed EGFR activating mutations, 2) treated with immune checkpoint inhibitors after disease progression with EGFR-TKIs. Patients without measurable tumour lesions or treated with immune checkpoint inhibitors in front line settings were excluded. We retrospectively reviewed the electric medical records of enrolled patients, and collected their detailed clinicopathologic characteristics and clinical responses. This study was approved by the institutional review board of Shandong Cancer Hospital and Institute and was performed in accordance with the Declaration of Helsinki.

### Treatment Procedures

All patients were treated with first-, second-, or third-generation EGFR-TKIs prior to immune checkpoint inhibitors. For immunotherapy, patients were treated with one of the following anti-PD-1 or PD-L1 agents until disease progression, or unacceptable toxicity: pembrolizumab (Merck & Co., USA), nivolumab (Bristol-Myers Squibb, USA), sintilimab (Innovent Biologics, China), toripalimab (Shangha Merck & Co.), camrelizumab (Jiangsu Hengrui Medicine, China), tislelizumab (BeiGene, China), durvalumab (AstraZeneca, USA), or atezolizumab (Roche, USA). This study was approved by the institutional review board of Shandong Cancer Hospital and Institute and was performed in accordance with the Declaration of Helsinki. Individual consent for this retrospective analysis was waived.

### Outcomes

Radiological assessments of primary and metastatic lesions were performed every 6 weeks during treatment. Therapeutic responses were evaluated with RECIST 1.1. Objective tumor responses included complete response (CR), partial response (PR), stable disease (SD), and progressive disease (PD). Progression-free survival (PFS) was defined as the time interval from the first-time administration of anti-PD-1 or PD-L1 agents to confirmed disease progression or mortality from any cause. Overall survival (OS) was defined as the time interval from the first-time administration of anti-PD-1 or PD-L1 agents to mortality from any cause or the last follow-up. Safety profiles were evaluated according to National Cancer Institute Common Terminology Criteria for Adverse Events, version 4.0.

### Statistical Analysis

All statistical analyses were performed using Statistical Product and Service Solutions (SPSS) version 26.0. For survival analyses, Kaplan-Meier analysis were performed, and log-rank test was used for comparison of survival times. Uni- and multi-variate Cox regression model were employed to analyse factors that may associated with treatment response and prognosis. Variates with *P* value <0.1 in univariate analyses were then subjected for multivariate analysis. In all analyses, differences were significant when *P* < 0.05.

## Results

### Patient Characteristics

In total, 75 patients were enrolled in this retrospective study. The relevant clinical and pathological characteristics are included in **Table 1**. Among the included patients, the distribution of male and female is relatively equal (49.3% *vs* 50.7%). The median age of patients were 52 years old, ranges from 36 to 81 years old. The majority of patients have stage IV disease. EGFR exon 21 L858R (40%) and 19 exon del (49.3%) are the most common mutation types. Gefitinib was the most commonly used EGFR-TKI in first-line setting (57.3%). During TKI treatment, 22 patients (29.3%) achieved partial regression, and 18 patients acquired subsequent T790M mutation. Most patients received immunotherapy in late line (≥3) setting. Thirty-one patients (41.3%) received immunotherapy concurrent with chemotherapy, 16 (21.3%) are treated together with antiangiogenic therapy, 24 patients (32%) received immunotherapy alongside with chemotherapy and antiangiogenic therapy, while only 4 patients (5.3%) received monotherapy. However, since immunotherapy was applied in late-line settings, PD-L1 expression was not reported in the majority (80%) of enrolled patients. During immunotherapy, 11 patients (14.7%) received concurrent radiotherapy, while 8 (10.7%) received extracranial radiotherapy.

**Table 1:** Patient characteristics.

Factor	N (%)
Gender	
Male	37 (49.3%)
Female	38 (50.7%)
Age (median, range) (y)	52.0 (36.0-81.0)
Smoking history (n)	
Never-smoker	63 (84.0%)
Former/current smoker	12 (16.0%)
T stage in naïve (n)	
1-2	40 (53.3%)
3-4	35 (46.7%)
TNM stage (n)	
IIIB-IIIC	4 (5.3%)
IVA-IVB	71 (94.7%)
Type of mutation (n)	
EGFR exon 19 del	30 (40%)
EGFR exon 21 L858R	37 (49.3%)
Others*	8 (10.7%)
Acquired T790M mutation	
No	57 (76.0%)
Yes	18 (24.0%)
Type of EGFR-TKI (n)	
Gefitinib	43 (57.3%)
Erlotinib	11 (14.7%)
Icotinib	10 (13.3%)
Afatinib	8 (10.7%)
Osimertinib	3 (4.0%)
Best response to EGFR-TKIs (n)	
PR	22 (29.3%)
SD/PD	53 (70.7%)
Number of immunotherapy lines (n)	
2	24 (32.0%)
≥3	51 (68.0%)
Treatment regimen (n)	
Anti-PD-1/PD-L1 monotherapy	4 (5.3%)
Anti-PD-1/PD-L1 + Chemo	31 (41.3%)
Anti-PD-1/PD-L1+Anti-angiogenesis	16 (21.3%)
Anti-PD-1/PD-L1+Chemo+anti-angiogenesis	24 (32.0%)
PD-L1 expression	
Negative	3 (4.0%)
1-49%	6 (8.0%)
≥50%	6 (8.0%)
Not reported	60 (80.0%)

^*^Others include EGFR exon 20INS (n=3), EGFR exon 18 G719A (n=2), EGFR exon 20 S768I (n=1), EGFR exon 21 L861Q (n=1), EGFR exon 21 G863D (n=1).

EGFR, Epidermal growth factor; TKI, Tyrosine kinase inhibitor; PR, Partial response; SD, Stable disease; PD, Progression disease.

### Efficacy of Immunotherapy

**Figure 1:**
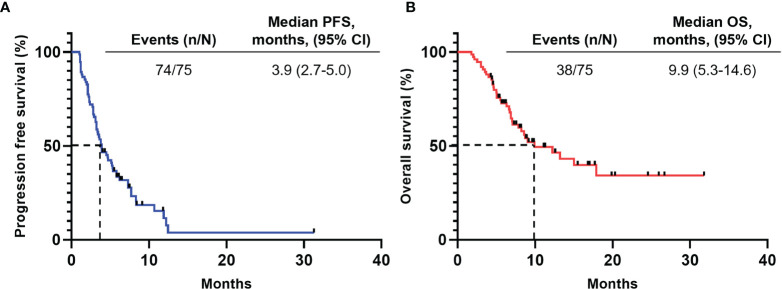
Survival outcomes. The median PFS for all patients was 3.9 months (95% CI, 2.7-5.0), while the median OS was 9.9 months (95% CI, 5.3-14.6). **(A)** Progression-free survival. **(B)** Overall survival. CI, Confidence interval.

**Figure 2:**
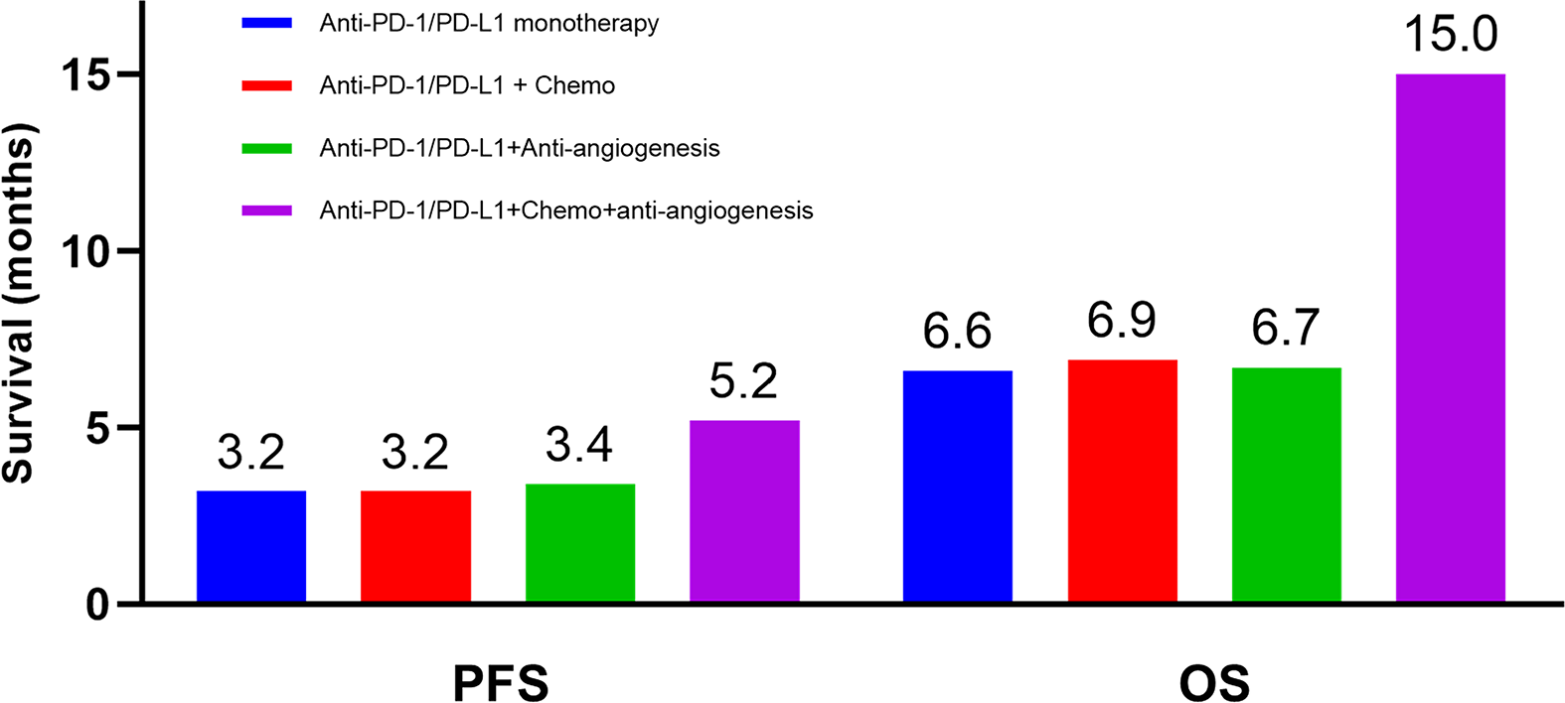
Median survival of patients with different therapeutic regimen. Blue: anti-PD-1/PD-L1 monotherapy, Red: anti-PD-1/PD-L1 plus chemotherapy, Green: anti-PD-1/PD-L1 plus anti-angiogenic agents, Purple: anti-PD-1/PD-L1 plus chemotherapy plus anti-angiogenic therapy. PFS, progression-free survival; OS, overall survival.

The median follow-up period was 7.3 months (range 1.8-31.8 months). During treatment, 6 patients (8.0%) achieved partial regression (PR), 53 patients (70.7%) experienced stable disease (SD), and 16 patients (21.3%) developed progression disease (PD), yielding an overall response rate (ORR) of 8.0%, and a disease control rate (DCR) of 78.7%. As shown in **Figure 1**, the median PFS for all patients was 3.9 months (95% CI, 2.7-5.0), while the median OS was 9.9 months (95% CI, 5.3-14.6). The comparison of the PFS and OS of different therapeutic regimen are displayed in **Figure 2**. Although did not reach statistical significance, patients received immunotherapy plus chemotherapy plus anti-angiogenic agents showed longest survival compared to other groups (median PFS 5.2 months, median OS 15.0 months), while patients received immune-monotherapy showed the shortest survival (median PFS 3.2 months, median OS 6.6 months).

As different type of primary EGFR mutation may interfere patient’s response to immunotherapy, we further analyzed the impact of different mutation types on survival. In general, we found no difference in PFS nor OS among different EGFR mutant (all *P*-value >0.05). However, across all treatment settings, although reach no statistical significance, longer OS were observed in patients bear L858R mutant, suggesting patients bear EGFR L858R mutant may benefit more from immunotherapy. For acquired resistance, no significant difference was found in both PFS and OS between patients with or without acquired T790M mutant (all *P*-value >0.05) (**Table 2**).

**Table 2:** Efficacy of immunotherapy in patients with different types of EGFR mutations.

	Mutation type	General (95%CI), mo	ICI alone (95%CI), mo	ICI+Chemo (95%CI), mo	ICI+Anti-angiogenesis (95%CI), mo	ICI+Chemo+Anti-angiogenesis (95%CI), mo
Primary	19del	PFS:3.87 (1.823-5.917) OS: 7.07 (4.394-9.746)	PFS:3.2 OS:6.63	PFS: 3.1 (1.396-4.806) OS: 9.93 (0.652-19.208)	PFS: 3.42 (1.829-5.011) OS: 4.67 (4.311-5.029)	PFS: 7.3 (3.839-10.761) OS: 7.83 (4.546-11.114)
	21 L858R	PFS: 3.9 (2.619-5.181) OS: 13.2 (5.497-20.903)	PFS: 4.7 (1.499-7.901) OS: 17.9 (0-43.073)	PFS: 5.31 (2.621-7.999) OS: 13.2 (7.018-19.382)	PFS: 3.1 (1.991-4.209) OS: 6.73 (3.958-9.502)	PFS: 3.9 (3.608-4.192) OS: 11.42 (8.971-13.874)
	Other	PFS: 2.3 (1.092-3.508) OS: 9.03	/	PFS: 1.4 (0.322-2.478) OS: 8.7 (3.957-13.443)	PFS: 5.8 OS: NR	PFS: 2.3 (0.22-4.380) OS: NR
Acquired	T790M-	PFS: 3.6 (2.129-5.071) OS: 8.7 (3.789-13.611)	PFS: 2.7 OS: 2.17	PFS: 3.1 (1.566-4.634) OS: 9.02 (5.354-12.706)	PFS: 3.1 (2.048-4.152) OS: 6.73 (1.593-3.607)	PFS: 4.67 (1.312-8.028) OS: NR
	T790M+	PFS: 3.9 (2.276-5.524) OS: 15 (4.884-25.116)	PFS: 3.2 OS: 6.63	PFS: 3.2 (1.16-5.24) OS: NR	PFS: 3.9 (0-9.453) OS: 5	PFS: 5.2 (2.436-7.964) OS: 15.0 (5.727-24.273)

CI, Confidence interval; ICI, Immune checkpoint inhibitor; PFS, Progression free survival; OS, Overall survival; NR, Not reached.

### Factors Associated With Therapeutic Efficacy of Immunotherapy

**Figure 3:**
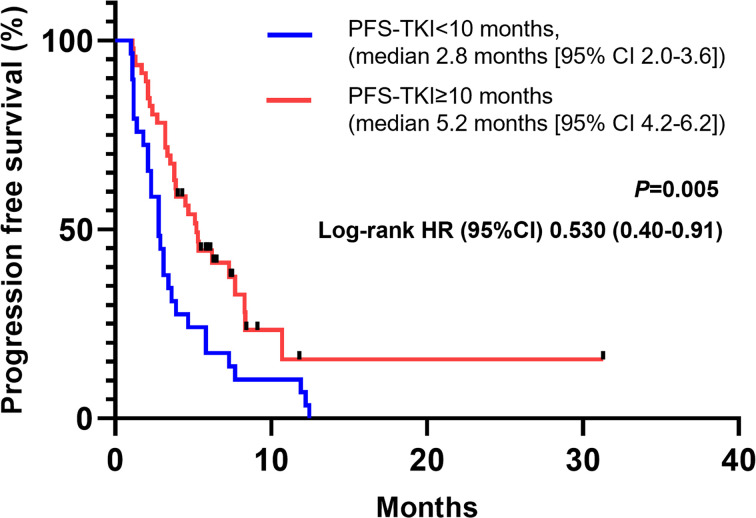
Patients with PFS-TKI longer than 10 months showed longer PFS during immunotherapy. CI, confidence interval; HR, hazard ratio.

According to previous report, the PFS of front-line EGFR-TKI may predict the therapeutic efficacy of posterior immunotherapy. In our study cohort, the median PFS of EGFR-TKI was 10 months (95% CI, 8.69-11.31). Similar to previous report, we found a cut-off value at 10 months would achieve most statistical differences in predicting the PFS of immunotherapy. As displayed in **Figure 3**, patients with longer PFS-TKI (≥10 months) showed a longer PFS when treated with immunotherapy compared with patients with shorter PFS-TKI (<10 months), the median PFS in two groups were 5.2 months [95%CI 4.2-6.2] and 2.8 months [2.0-3.6]) respectively (HR, 0.53, 95%CI, 0.31-0.91, *P*=0.005).

**Figure 4:**
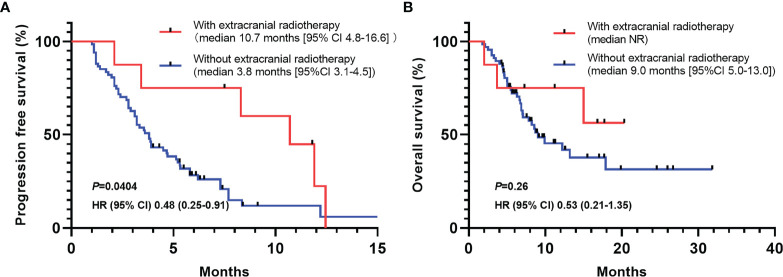
PFS and OS of patients with or without concurrent extracranial radiotherapy during immunotherapy. **(A)** Patients received concurrent extracranial radiotherapy showed significantly longer PFS (HR, 0.48, 95%CI 0.25-0.91, *P*=0.0404). **(B)** Overall survival of patients with or without concurrent extracranial radiotherapy. HR, hazard ratio; CI, confidence interval.

As more and more studies have demonstrated that concurrent radiotherapy during immunotherapy plays an essential role in inflaming immune microenvironment and improve the efficacy of immunotherapy, we perform a sub-group analysis to study the effect of extracranial radiotherapy on the efficacy of immunotherapy. We found that concurrent extracranial radiotherapy is associated with longer PFS (median PFS, 10.7 months [95% CI 4.8-16.6] *vs* 3.8 months [3.1-4.5]) (HR, 0.48, 95%CI 0.25-0.91, *P*=0.0404) (**Figure 4A**). Although have not reached statistical significance, patients with concurrent extracranial radiotherapy also showed longer OS (median OS, NR *vs* 9.0 months [95%CI 5.0-13.0]) (HR, 0.53, 95%CI 0.21-1.35, *P*=0.26) (**Figure 4B**).

**Figure 5:**
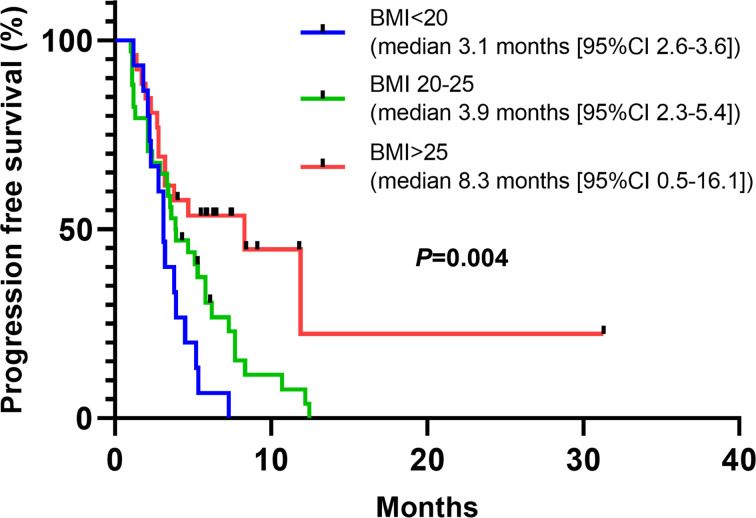
Patients with higher BMI are associated with better response to immunotherapy. Patients with BMI higher than 25 showed longer PFS compared with patients with lower BMI (*P*=0.004). BMI, body mass index.

We further performed uni- and multi-variate Cox Regression Analysis to explore the potential clinical and pathological parameters that may be associated with PFS or OS. In consistent with our previous results, in multivariate cox analysis, we found that PFS-TKI and concurrent extracranial radiotherapy are associated with PFS (*P* values are 0.006 and 0.021 respectively) (**Table 3**). No parameters were found related to OS (**Table 4**). Interestingly, we found body mass index was associated with PFS in univariate cox analysis. Linear regression revealed that larger BMI is associated with longer PFS (r=0.4, *P*=0.005), further subgroup analysis also indicated that patients with BMI over 25 is associated with longer PFS (*P*=0.004) (**Figure 5**).

**Table 3:** Uni- and multivariate Cox regression analysis of factors associated with PFS.

Factors	Univariate analysis		Multivariate analysis	
	HR (95% CI)	*p* Value	HR (95% CI)	*p* Value
Gender				
Male				
Female	1.061 (0.638-1.765)	0.818		
Age				
<60				
≥60	1.473 (0.833-2.605)	0.183		
Body mass index				
≤20		**0.007**		0.126
20-25	3.202 (1.509-6.793)	**0.002**	2.780 (0.957-8.074)	0.060
≥25	1.97 (1.046-3.711)	**0.036**	2.461 (0.947-6.396)	0.065
T stage				
T1-2				
T3-4	0.945 (0.569-1.570)	0.828		
Presence of liver metastasis				
Yes				
No	1.433 (0.723-2.842)	0.303		
Presence of brain metastasis				
Yes				
No	0.939 (0.536-1.647)	0.827		
Types of mutation				
Exon 19 del		0.374		
Exon 21 L858R	0.562 (0.247-1.277)	0.169		
Others	0.605 (0.272-1.347)	0.605		
Types of EGFR-TKI				
Gefitinib		0.444		
Erlotinib	1.450 (0.347-6.051)	0.611		
Icotinib	1.838 (0.395-8.549)	0.438		
Afatinib	1.373 (0.289-6.518)	0.690		
Osimertinib	2.891 (0.601-13.911)	0.185		
Acquired T790M mutation				
Yes				
No	0.893 (0.421-1.894)	0.797		
PFS to EGFR-TKIs				
<10 months				
≥10 months	1.934 (1.155-3.237)	**0.012**	5.279 (1.629-17.114)	**0.006**
Previous extracranial radiotherapy				
Yes				
No	0.944 (0.558-1.595)	0.828		
Previous thoracic radiotherapy				
Yes				
No	0.981 (0.557-1.729)	0.948		
Number of immunotherapy lines (n)				
2				
≥3	1.352 (0.823-2.220)	0.234		
Treatment regimen (n)				
Anti-PD-1/PD-L1 monotherapy		0.488		
Anti-PD-1/PD-L1 + Chemo	0.807 (0.233-2.793)	0.734		
Anti-PD-1/PD-L1+Anti-angiogenesis	1.379 (0.747-2.546)	0.304		
Anti-PD-1/PD-L1+Chemo+anti-angiogenesis	1.572 (0.788-3.138)	0.199		
Concurrent extracranial radiotherapy				
Yes				
No	2.251 (0.936-5.417)	0.070	4.694 (1.266-17.406)	**0.021**

CI, Confidence interval; PFS, Progression free survival; EGFR, Epidermal growth factor; TKI, Tyrosine kinase inhibitor. Bold: P value < 0.05.

**Table 4:** Uni- and multivariate Cox regression analysis of factors associated with OS.

Factors	Univariate analysis		Multivariate analysis	
	HR (95% CI)	*p* Value	HR (95% CI)	*p* Value
Gender				
Male				
Female	0.955 (0.501-1.822)	0.890		
Age				
<60				
≥60	0.715 (0.353-1.449)	0.352		
Body mass index				
≤20		0.354		
20-25	1.359 (0.515-3.586)	0.535		
≥25	1.745 (0.815-3.739)	0.152		
T stage				
T1-2				
T3-4	1.226 (0.639-2.354)	0.540		
Presence of liver metastasis				
Yes				
No	0.592 (0.260-1.353)	0.214		
Presence of brain metastasis				
Yes				
No	0.593 (0.304-1.156)	0.125		
Types of mutation				
Exon 19 del		0.657		
Exon 21 L858R	1.588 (0.462-5.458)	0.463		
Others	1.231 (0.362-4.182)	0.739		
Acquired T790M mutation				
Yes				
No	1.242 (0.585-2.640)	0.573		
PFS to EGFR-TKIs				
<10 months				
≥10 months	1.610 (0.841-3.083)	0.150		
Previous extracranial radiotherapy				
Yes				
No	0.948 (0.489-1.838)	0.875		
Previous thoracic radiotherapy				
Yes				
No	0.723 (0.368-1.421)	0.347		
Number of immunotherapy lines (n)				
2				
≥3				
Treatment regimen (n)				
Anti-PD-1/PD-L1 monotherapy		0.727		
Anti-PD-1/PD-L1 + Chemo	1.583 (0.420-5.970)	0.498		
Anti-PD-1/PD-L1+Anti-angiogenesis	1.424 (0.623-3.255)	0.402		
Anti-PD-1/PD-L1+Chemo+anti-angiogenesis	1.658 (0.666-4.124)	0.277		
Concurrent extracranial radiotherapy				
Yes				
No	1.919 (0.578-6.371)	0.287		

CI, Confidence interval; PFS, Progression free survival; EGFR, Epidermal growth factor; TKI, Tyrosine kinase inhibitor.

## Discussion

In this retrospective study, we examine the therapeutic efficacy of ICIs alone or in combination in patients with EGFR mutated NSCLC, founding that a combination of ICIs and anti-angiogenic agents plus chemotherapy would lead to longer PFS and OS, while safety profile is tolerable. For the first time, we also found that concurrent extracranial radiotherapy would significantly prolong the PFS of ICI treatment. We also found that longer PFS-TKI and larger BMI could be a predictor for better response to immunotherapy.

Patients bear EGFR mutations has long been associated with inferior response to second and late line chemotherapy after resistance to EGFR-TKI. In IMPRESS trial, median PFS for patients receiving chemotherapy after resistant to first line gefitinib was 5.4 months, while in AURA3 study, a median PFS of 4.4 months were reported in patients receiving platinum-based chemotherapy after acquired T790M mutation following gefitinib resistance (15, 16). In our study, the median PFS is 3.9 months, lower compared to historical controls. However, as the majority of enrolled patients were treated with immunotherapy in late (3+) line settings, these results could not be compared directly. In recent study, Yu et al. showed that after fail to EGFR-TKI treatment, patients received ICIs plus chemotherapy as second-line therapy had higher response rate compared to anti-angiogenesis plus chemotherapy (ORR 29.5% vs. 13.0%, *P*=0.018), but no significant difference in patient’s prognosis (median PFS 7.59 vs. 6.90 months, *P*=0.552) *(*17). Hence, the value of second-line immunotherapy in patients fail EGFR-TKI treatment requires further investigation.

Studies in EGFR mutated murine models and cell lines suggested that the activation of EGFR would up-regulate PD-L1 expression, and anti-PD-1 therapy could improve the survival of mice with EGFR mutated tumors. However, further studies of clinical samples imply that patients harbor EGFR mutations are associated with fewer infiltrated immune cells and lower PD-L1 expression level, therefore an “immune-cold” microenvironment (18, 19). In practice, patients with EGFR mutations are usually associated with inferior response to anti-PD-1/PD-L1 monotherapy. Although pre-clinical studies suggest that treatment of EGFR-TKIs would inflaming immune microenvironment *via* improve T cell infiltration and decrease the infiltration of CD4+ regulatory T cells (6, 20, 21), further clinical trials showed that combination of EGFR-TKIs and anti-PD-1 therapy lead to an unacceptable occurrence rate of adverse events, especially interstitial pneumonitis (10, 22).

In consistent with our study, clinical trials and real-world data indicated that combination of ICIs with chemotherapy plus anti-angiogenic agents would yield longer survival comparing to ICI monotherapy or ICIs plus either chemotherapy or anti-angiogenic therapy alone. In IMpower150 study, 58 patients with sensitizing EGFR mutation were enrolled, and the median overall survival were significantly prolonged in patients received ABCP (atezolizumab plus bevacizumab plus chemotherapy), compared to patients received ACP (atezolizumab plus chemotherapy) regimen (11). Numbers of clinical trials have demonstrated that combination of bevacizumab and EGFR-TKI would provide clinical benefits to patients with sensitive EGFR mutations, comparing to EGFR-TKI alone, indicating the value of anti-angiogenic therapy in this patient cohort (23, 24). In mechanism, activation of EGFR signaling pathway in tumor would up-regulate VEGF expression, hence sensitize to anti-angiogenic therapies (25).

Although did not met statistical significance, our study suggested that patients bear EGFR L858R mutation may benefit more from immunotherapy, both alone and in combination. This result is consisted with previous reports that patients bear EGFR L858R mutation have a higher response rate compared to patients bear 19del mutation (13). In mechanism, tumors with L8585R mutation have higher level of tumor mutation burden (TMB) compared to tumors with 19del mutation. Recent study also suggested that tumor with EGFR L858R mutation have higher level of PD-L1 expression and are positively associated with inflammatory phenotype (26).

The relationship between the therapeutic efficacy of EGFR-TKIs in front lines and ICIs in late lines are still in debate. In our study, we found that patients with longer response duration to EGFR-TKIs tends to have longer PFS in immunotherapy, with median PFS of 5.2 months *vs* 2.8 months. In contrast with our finding, a retrospective study by Liu et al. demonstrated that patients with shorter PFS to EGFR-TKI are associated with better response to late line immunotherapies (14). As treatment of EGFR-TKIs may inflame immune microenvironment by promoting the release of neoantigens, infiltration of effector T cells, and production of pro-inflammatory cytokines, we hypothesis that longer TKI treatment period may transform the original “cold” immune microenvironment to a “hotter” one, therefore more suitable for ICIs. However, the inconsistency of results may also cause by limited sample size and tumor heterogeneity, and requires further investigation in larger patient populations.

Radiotherapy may work synergistically with immunotherapy, preclinical evidences indicated that radiotherapy may re-programme tumor microenvironment by promoting the release of tumor neoantigen, activating innate immune response *via* cGAS/STING pathway and improve immune cell infiltration. A reanalysis of KEYNOTE-001 trial indicated that patients with advanced NSCLC who received previous radiotherapy would yield longer progression-free survival and overall survival with pembrolizumab treatment comparing to patients without prior radiotherapy (27). In a recent pooled analysis of 2 major clinical trials that evaluate the therapeutic efficacy of pembrolizumab with or without radiotherapy in patients with metastatic NSCLC, Theelen et al. found that the patients treated concurrently with pembrolizumab and radiotherapy result in higher response rate as well as longer PFS and OS (28). However, the role of radio-immunotherapy in patients with EGFR mutations is still unclear (29). In our study, for the first time, we reported that concurrent extracranial radiotherapy during immunotherapy is associated to longer PFS (10.7 months *vs* 3.8 months), and although did not meet statistical significance, patients received radiotherapy plus immunotherapy also showed longer OS (NR *vs* 9.0 months). However, due to very limited sample size, the role of radio-immunotherapy in patients with EGFR mutation still requires further investigation.

Interestingly, in multivariate Cox regression, we found that larger BMI is associated with longer PFS. There is a complicated relationship between obesity and cancer prognosis, obesity may increase the risk of cancer development, but can also protect patients with advanced NSCLC from worse outcomes, such as wasting (30). In a pooled analysis of 4 clinical trials that compared the efficacy of atezolizumab versus docetaxel in patients with advanced NSCLC, Kichenadasse et al. found that higher BMI is independently associated with better prognosis with atezolizumab, especially in patients with high expression of PD-L1 (31). In mechanism, obese adipose tissue is regarded as chronically inflammation tissues, through expressing inflammatory cytokines such as IL-1beta, IL-6, IL-10, and TNF-alpha, cancer-associated obese adipocyte recruits macrophages, neutrophils and other immune cells into tumour microenvironment (32). Moreover, by producing leptin, obesity also impairs the function of T cells, increasing the proportion of exhausted PD-1 positive T cell (33, 34). Hence, the association between higher BMI and better response to immunotherapy is probably due to the existence of exhausted PD-1 positive T cells in adipose tissue.

In conclusion, in our study, we found that combination regimen of immunotherapy plus chemotherapy plus anti-angiogenic therapy would yield better PFS and OS in patients with EGFR mutations. We also found that longer response duration to EGFR-TKIs, concurrent extracranial radiotherapy, and higher BMI are independently associated with better response to immunotherapy. However, due to a relatively low sample size (n=75), these conclusions still require further validation in a larger patient population.

## Data Availability Statement

The original contributions presented in the study are included in the article/**Supplementary Material**. Further inquiries can be directed to the corresponding authors.

## Ethics Statement

The studies involving human participants were reviewed and approved by Institutional review board of Shandong Cancer Hospital and Institute. The ethics committee waived the requirement of written informed consent for participation.

## Author Contributions

MB, conceptualization, methodology, data curation, visualization, and writing - original draft. WW, data curation and visualization. XG and PJ, visualization and investigation. LW, data curation, writing- reviewing and editing. HW, data curation. JY, conceptualization and supervision. XM, conceptualization, writing- reviewing and editing, and supervision. All authors contributed to the article and approved the submitted version.

## Funding

This study was supported by National Natural Science Foundation of China (81972864), Academic Promotion Program of Shandong First Medical University (2019RC002), Science and Technology Support Plan for Youth Innovation Teams of Universities in Shandong Province (2019KJL001), Science and Technology Plan of Jinan (201907113), Medical Science and Technology Project of Henan Province (SB201901112).

## Conflict of Interest

The authors declare that the research was conducted in the absence of any commercial or financial relationships that could be construed as a potential conflict of interest.

## Publisher’s Note

All claims expressed in this article are solely those of the authors and do not necessarily represent those of their affiliated organizations, or those of the publisher, the editors and the reviewers. Any product that may be evaluated in this article, or claim that may be made by its manufacturer, is not guaranteed or endorsed by the publisher.
